# IGFBPL1 inhibits macrophage lipid accumulation by enhancing the activation of IGR1R/LXRα/ABCG1 pathway

**DOI:** 10.18632/aging.205301

**Published:** 2023-12-28

**Authors:** Lianjie Hou, Xixi Feng, Zhi Zhu, Yali Mi, Qin He, Kai Yin, Guojun Zhao

**Affiliations:** 1 The Sixth Affiliated Hospital of Guangzhou Medical University, Qingyuan City People's Hospital, Qingyuan 511518, Guangdong, China; 2 Guangzhou Huali Science and Technology Vocational College, Guangzhou 511325, Guangdong, China; 3 Dali University, Dali 671003, Yunnan, China; 4 Department of Cardiology, The Second Affiliated Hospital of Guilin Medical University, Guilin 541001, Guangxi, China

**Keywords:** insulin-like growth factor 1, lipid accumulation, macrophages, cholesterol efflux, atherosclerosis

## Abstract

Lipid accumulation in macrophages plays an important role in atherosclerosis and is the major cause of atherosclerotic cardiovascular disease. Reducing lipid accumulation in macrophages is an effective therapeutic target for atherosclerosis. Insulin-like growth factor 1 (IGF-1) exerts the anti-atherosclerotic effects by inhibiting lipid accumulation in macrophages. Furthermore, almost all circulating IGF-1 combines with IGF binding proteins (IGFBPs) to activate or inhibit the IGF signaling. However, the mechanism of IGFBPs in macrophage lipid accumulation is still unknown. GEO database analysis showed that among IGFBPS family members, IGFBPL1 has the largest expression change in unstable plaque. We found that IGFBPL1 was decreased in lipid-laden THP-1 macrophages. Through oil red O staining, NBD-cholesterol efflux, liver X receptor α (LXRα) transcription factor and IGR-1 receptor blocking experiments, our results showed that IGFBPL1 inhibits lipid accumulation in THP-1 macrophages through promoting ABCG1-meditated cholesterol efflux, and IGFBPL1 regulates ABCG1 expression and macrophage lipid metabolism through IGF-1R/LXRα pathway. Our results provide a theoretical basis of IGFBPL1 in the alternative or adjunct treatment options for atherosclerosis by reducing lipid accumulation in macrophages.

## INTRODUCTION

Cardiovascular disease caused by atherosclerosis is a major cause of morbidity and mortality worldwide [1]. Numerous studies have elucidated that abnormal level of blood lipid is closely related to the occurrence of atherosclerosis [2]. Macrophages plays a critical role in regulating the lipid level in blood and tissue [3]. Macrophages uptake modified low-density lipoprotein and account for lipid accumulation [4]. Then the macrophages deposit in the arterial wall, resulting in narrowing of the vessels and atherosclerotic lesions [5]. Therefore, reducing lipid accumulation in macrophages is an effective therapeutic strategy for treating atherosclerosis and has always attracted the attention of scientists.

IGF-1, the insulin superfamily member, is the most abundant and ubiquitous polypeptide growth factor, and plays pleiotropic roles in regulating cell function. There are growing evidence indicating that IGF-1 plays an important role in the development of atherosclerosis [6, 7]. The decreasing of IGF-1 in serum is associated with high cardiovascular disease risk, and IGF-1 administration reduces atherosclerosis in mice with apolipoprotein E knockout [8]. The anti-atherosclerotic effect of IGF-1 is partly through inhibiting lipid accumulation in macrophages [9].

About 99% IGF-1 is bound to IGF binding proteins (IGFBPs) and then regulate the IGF signaling pathway [10] IGFBP like protein1 (IGFBPL1) is a member of IGFBPs family [11, 12]. The human IGFBPL1 gene located on chromosome 9p13.1, contains 6 exons to coding a protein containing 278 amino acids. Like other family members, IGFBPL1 regulates cell IGF signal by binding with IGFs [13]. In mice, the neural stem/precursor cells in the sub-ventricular zone protect against the impairment of striatal medium spiny neuron morphology and the dysfunctions of striatum-related behavior by secreting IGFBPL1 [11]. In breast cancer, the expression of IGFBPL1 is regulated by aberrant hypermethylation, implying that the down-regulation of IGFBPL1 is involved in the pathogenesis of this malignancy [13]. In esophageal cancer, IGFBPL1 suppressed human esophageal cancer cell xenografts growth in mice by inhibiting esophageal cancer cell proliferation and inducing cell apoptosis [12]. However, there is no published article reporting the function of IGFBPL1 on lipid accumulation in macrophages and atherosclerosis. Through the Gene Expression Omnibus (GEO) database analysis, we found that IGFBPL1 was the top 10 down-regulated gene in unstable atherosclerosis plaque. Thus, this study will clarify the effect and mechanism of IGFBPL1 on the lipid accumulation in macrophages and provide a theoretical basis of IGFBPL1 in the treatment for atherosclerosis.

## RESULTS

### IGFBPL1 inhibits PA-induced lipid accumulation in macrophages

**Figure 1:**
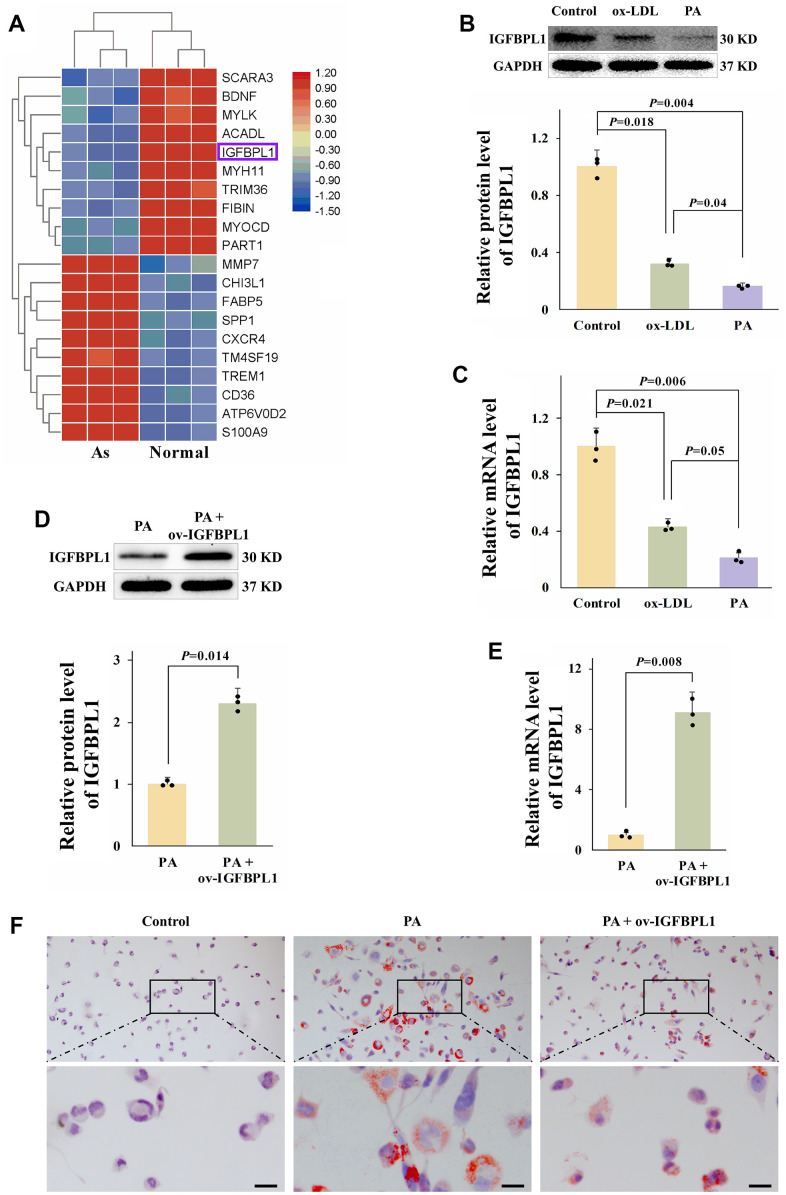
**IGFBPL1 inhibits PA-induced lipid accumulation in macrophages.** (**A**) Different expression gene in control samples and unstable atherosclerosis plaque human. Normal: the abdominal aortas intima of three patients without atherosclerosis. Atherosclerosis: aortic plaques of patients diagnosed with grade V or VI atherosclerosis. (**B**) Relative IGFBPL1 protein level (n=3). (**C**) Relative mRNA level of IGFBPL1 in PA or ox-LDL treated THP-1 macrophages (n=3). (**D**) Relative protein level of IGFBPL1 (n=3). (**E**) Relative IGFBPL1 mRNA level (n=3). (**F**) Representative images of oil red O staining. PA: palmitic acid, THP-1 macrophages were incubated with 100 μM PA for 48 h. ox-LDL: oxidized low density lipoprotein, THP-1 macrophages were treated with 100 μg/mL ox-LDL for 72 h. PA + ov-IGFBPL1, THP-1 was transfected with over-expression plasmid of IGFBPL1 for 24 h and then incubated with 100 μM PA for 48 h. *P*<0.05 represents a significant statistical difference; Scale indicates 25 μm.

To identify the candidate IGFBPs potentially related to atherosclerosis and macrophages lipid accumulation, we downloaded the microarray dataset GSE97210 from the Gene Expression Omnibus (GEO) database, and the different genes were screen by GEO2R. The top 20 differently expressed genes from GSE97210 showed that IGFBPL1 was decreased in unstable atherosclerosis plaque (Figure 1A). We also detected the IGFBPL1 protein level in lipid-laden THP-1 macrophages. The WB result showed that IGFBPL1 protein level was significantly reduced in palmitic acid (PA) or oxidized low density lipoprotein (ox-LDL) induced lipid-laden THP-1 macrophages (Figure 1B). We also assessed IGFBPL1 mRNA content in lipid-laden THP-1 macrophages. The qPCR result indicated that IGFBPL1 mRNA content was also decreased in lipid-laden THP-1 macrophages (Figure 1C).

To detect the function of IGFBPL1 on lipid accumulation, THP-1 macrophages were transfected with IGFBPL1 over-expression plasmid (ov-IGFBPL1). The qPCR and WB results indicated that IGFBPL1 overexpression plasmid up-regulated the mRNA and protein levels of IGFBPL1 in THP-1 macrophages (Figure 1D, 1E). Compared with the empty plasmid pcDNA3.1, the lipid droplets were decreased in macrophages transfected with IGFBPL1 plasmid, as showed by oil red O staining (Figure 1F). These results indicate that IGFBPL1 alleviates PA-induced lipid accumulation in THP-1 macrophage.

### IGFBPL1 inhibits lipid accumulation in macrophages by promoting ABCG1-meditated cholesterol efflux

**Figure 2:**
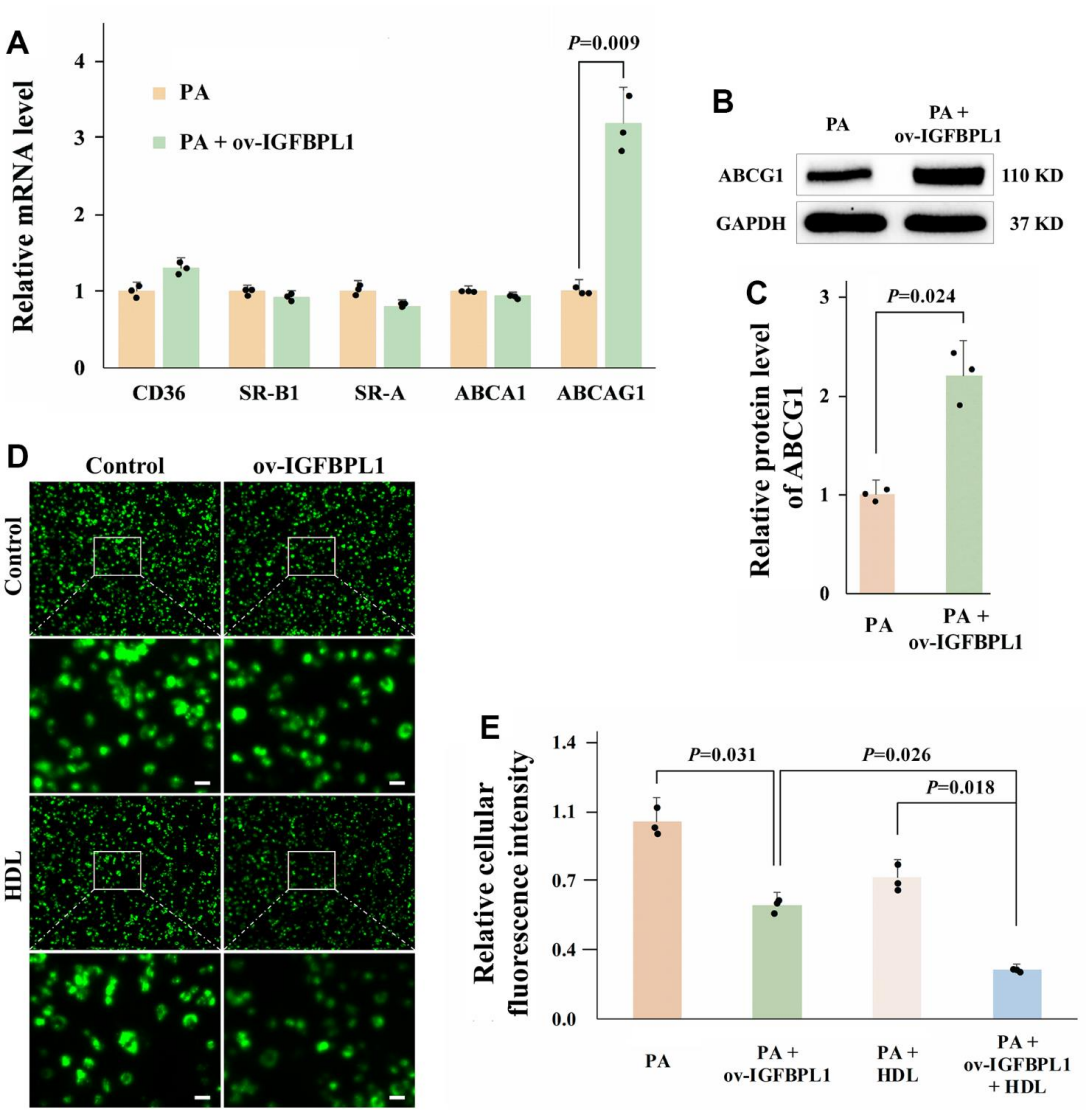
**IGFBPL1 increases macrophage cholesterol efflux through ABCG1.** (**A**) Relative mRNA level of genes relative to cholesterol uptake and efflux (n=3). (**B**) WB was used to detect the protein level of ABCG1. (**C**) Quantitative statistics on WB results by ImageJ (n=3). (**D**) Representative images of NBD cholesterol fluorescence staining of macrophages. (**E**) Quantitative results of average fluorescence intensity of per THP-1 macrophage cell (n=3). PA: palmitic acid, THP-1 macrophages were incubated with 100 μM PA for 48 h. PA + ov-IGFBPL1, THP-1 was transfected with over-expression plasmid of IGFBPL1 for 24 h and then incubated with 100 μM PA for 48 h. *P*<0.05 represents a significant statistical difference; Scale indicates 25 μm.

Macrophage lipid accumulation is related to the imbalance of cholesterol uptake and efflux [14]. The scavenger receptors (SR-A and CD36) are mainly responsible for uptake of lipoprotein-derived cholesterol. While the cholesterol efflux is meditated by ATP-binding cassette transporters A1(ABCA1), ABCG1, and SR-BI [15]. As shown by qPCR results, IGFBPL1 overexpression only increased ABCG1 mRNA levels, but did not change the mRNA levels of CD36, SR-A, SR-B1, and ABCA1 (Figure 2A). The WB result also showed that the protein level of ABCG1 was increased in THP-1 macrophages with IGFBPL1 overexpression (Figure 2B, 2C). ABCG1 reduces lipid accumulation in macrophages by mediating cholesterol efflux from macrophages to the extracellular cholesterol acceptor, HDL. Therefore, we observed the effect of IGFBPL1 on macrophage cholesterol efflux in the presence or absence of HDL through NBD labeled cholesterol. Fluorescence staining results showed that IGFBPL1 increased cholesterol efflux from macrophages, and the regulatory effect of IGFBPL1 on cholesterol efflux was further enhanced in the presence of HDL, suggesting that IGFBPL1 promoted cholesterol efflux from macrophages through ABCG1 (Figure 2D, 2E). These results indicate that IGFBPL1 inhibits macrophage lipid accumulation by increasing ABCG1-meditated cholesterol efflux.

### IGFBPL1 regulates ABCG1 expression and macrophage lipid accumulation through transcription factor LXRα

**Figure 3:**
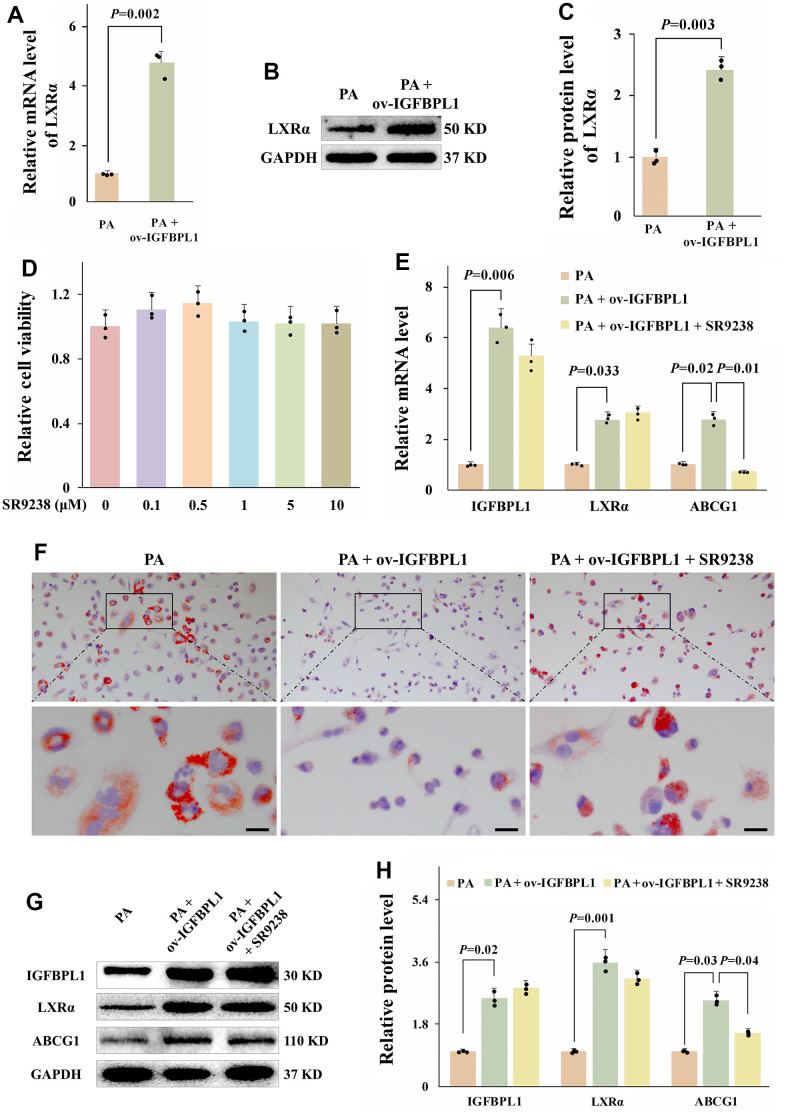
**LXR meditated the regulation effect of IGFBPL1 on ABCG1 expression and macrophage lipid accumulation.** (**A**) Relative mRNA level of LXR (n=3). (**B**) WB was used to detect the protein level of LXR. (**C**) Quantitative statistics on WB results by ImageJ (n=3). (**D**) CCK-8 was used to detect the cell viability (n=3). (**E**) Relative mRNA level of IGFBPL1, IGBPL1, LXR and ABCG1 (n=3). (**F**) Representative images of oil red O staining. (**G**) WB was used to detect the protein level of IGFBPL1, LXR and ABCG1. (**H**) Quantitative statistics on WB results by ImageJ (n=3). PA: palmitic acid, SR9238: LXRα antagonist. PA + ov-IGFBPL1 + SR9238, T THP-1 was transfected for 24 h and then incubated with PA (100 μM) and SR9238 (1 μM) for 48 h. *P*<0.05 represents a significant statistical difference; Scale indicates 25 μm.

ABCG1 expression is transcriptionally activated by LXRα. As shown by qPCR results, IGFBPL1 overexpression increased LXRα mRNA levels (Figure 3A). The WB result also showed that the protein level of LXRα was increased in THP-1 macrophages with IGFBPL1 overexpression (Figure 3B, 3C). To confirm that LXRα mediates the regulation of IGFBPL1 on lipid accumulation and ABCG1 expression in macrophages, we blocked LXRα transcription activity with SR9238, an LXRα antagonist. SR9238 inhibits the physiological function of LXRα without changing LXRα mRNA and protein levels. CCK-8 result showed that SR9238 had no obvious toxicity to THP-1 macrophages with the concentration lower than 10 μM (Figure 3D). Based on previous reports, 1 μM SR9238 was used in this research. Oil red O staining showed SR9238 reversed the IGFBPL1 inhibitory effect on THP-1 macrophage lipid accumulation (Figure 3F). The qPCR and WB results also showed that SR9238 reversed the up-regulation of ABCG1 induced by IGFBPL1 (Figure 3E, 3G, 3H). These results indicate that the regulation effect of IGFBPL1 on ABCG1 expression and macrophage lipid accumulation through LXRα.

### IGF-1R mediates the anti-lipid accumulation effect of IGFBPL1 in macrophages

**Figure 4:**
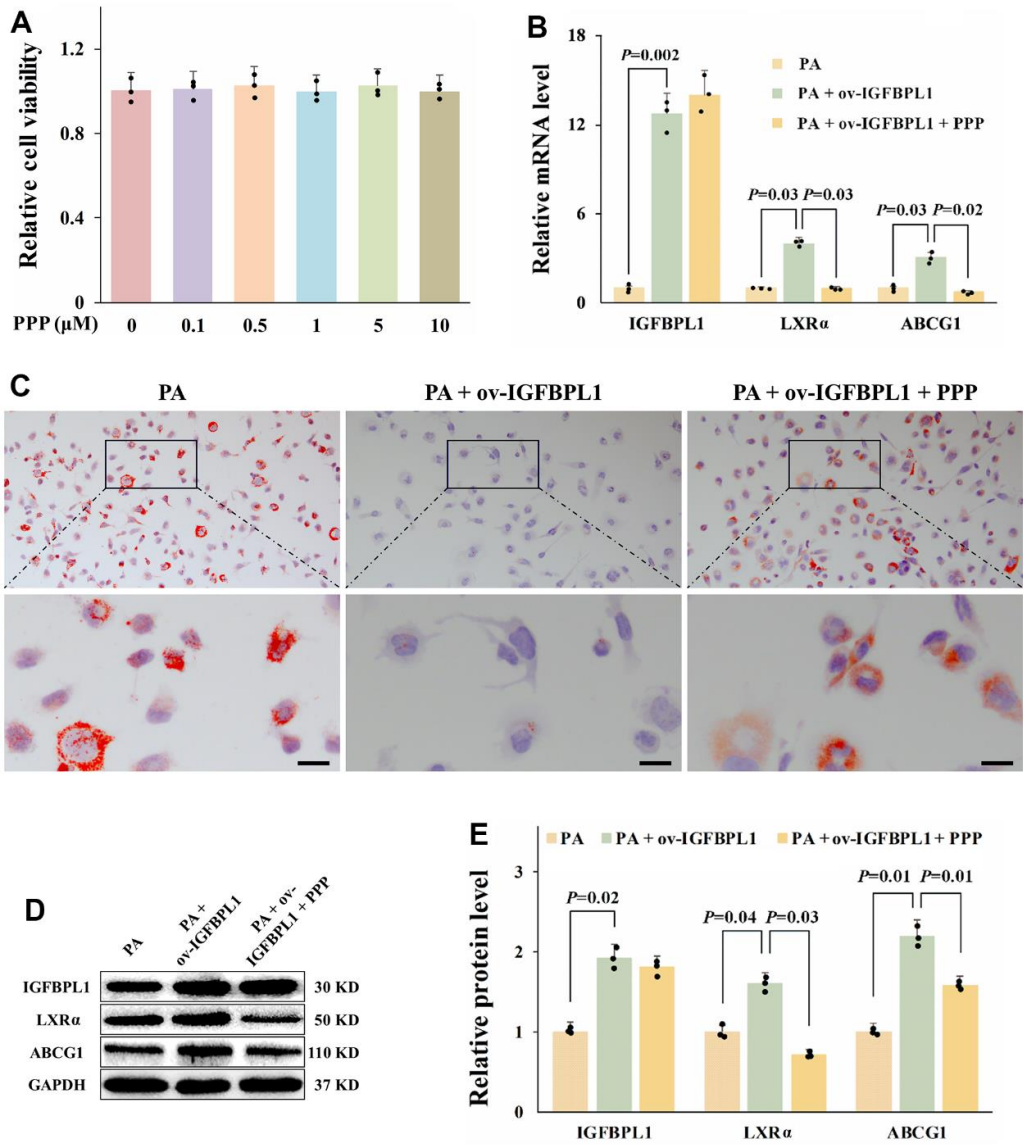
**IGF-1R is critical for the anti-lipid accumulation effect of IGFBPL1 in macrophages.** (**A**) Cell viability of THP-1 macrophages treated with PPP was detected by CCK-8. (**B**) Relative mRNA level of IGFBPL1, LXR and ABCG1 in THP-1 macrophages. (**C**) Representative images of oil red O staining. (**D**) WB was used to detect the protein level of LXR, ABCG1 and IGFBPL1 (n=3). (**E**) Quantitative statistics on WB results by ImageJ (n=3). PA: palmitic acid, PPP: picropodophyllin. PA + ov-IGFBPL1 + PPP, THP-1 was transfected for 24 h and then incubated with PA (100 μM) and PPP (1 μM) for 48 h. *P*<0.05 represents a significant statistical difference; Scale indicates 25 μm.

In addition to regulating IGF bio-activity, IGFBPs modulates cell biological processes by binding to a variety of extracellular and cell surface molecules directly [16]. To confirm whether the regulatory effect of IGFBPL1 on lipid accumulation and ABCG1 expression depends on IGF-1, we blocked IGF-1R through picropodophyllin to observe the changes of lipid accumulation and ABCG1 expression. CCK-8 result showed that PPP had no obvious toxicity to THP-1 macrophages with the concentration lower than 10 μM (Figure 4A). The oil red O staining result showed that PPP blocked the inhibitory effect of IGFBPL1 on the lipid accumulation of THP-1 macrophages (Figure 4C). qPCR and WB results showed that the PPP reversed the up-regulation of LXRα and ABCG1 mRNA and protein induced by IGFBPL1, but had no effect on IGFBPL1 expression (Figure 4B, 4D, 4E). These results indicate that the inhibitory effect of IGFBPL1 on lipid accumulation depends on IGF-1R.

## DISCUSSION

**Figure 5:**
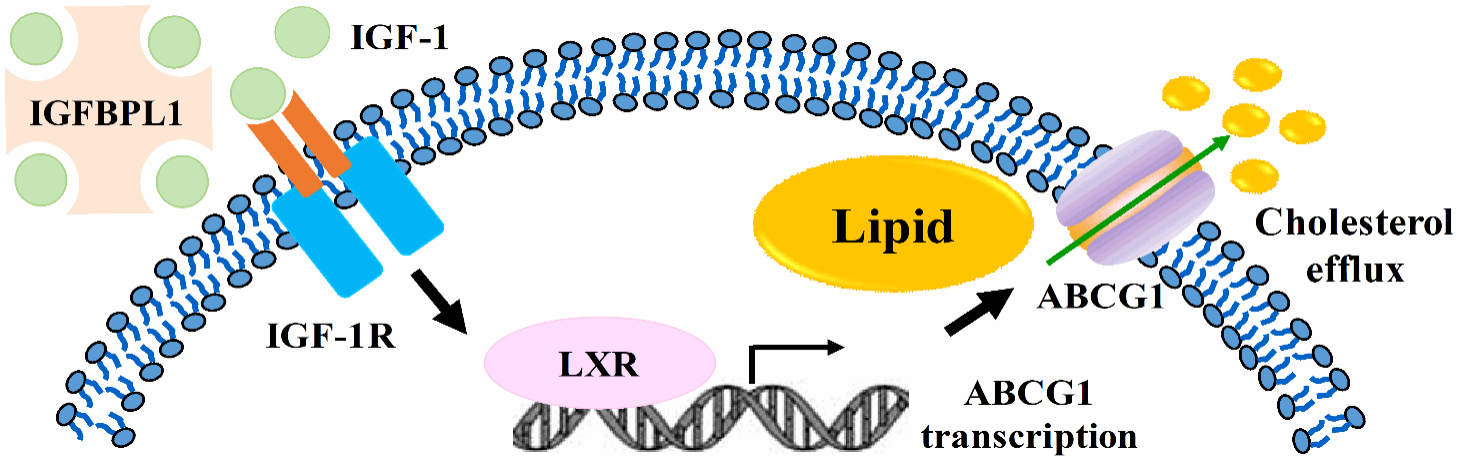
Summary model of IGFBPL1 inhibits macrophage lipid accumulation.

Atherosclerosis is a progressive and systemic disease with the character of inflammation response, lipid accumulation, and plaque formation in vessel wall [17]. The lipid-loaded macrophages formation and retention in the vessel wall accelerates the vulnerable plaques development [18]. Thus, identifying potential treatment options to decrease macrophage lipid accumulation is urgent. In this study, our results showed that the expression of IGFBPL1 was down regulated in unstable atherosclerosis plaque and lipid-laden macrophages. Moreover, our results also showed that IGFBPL1 inhibits macrophage lipid accumulation by enhancing the activation of IGR-1R/LXRα/ABCG1 pathway (Figure 5), suggesting that targeting IGFBPL1 may be an effective therapy for atherosclerosis.

As a pleiotropic factor, IGF-1 circulates in blood to exert endocrine effect [14]. The serum IGF-1 reaches the highest level in the pubertal growth phase, and declining with age [15]. In fact, epidemiologic data have suggested that low IGF-1 level is an important predictor of coronary event in aged subject [16]. In atherosclerosis mice, it has been shown that decreasing of serum IGF-1 is correlated with a higher risk of atherosclerosis and vice versa, a higher serum IGF-1 level inhibits atherosclerosis [17]. IGFBPs are key regulators of IGF-1 bioavailability in cell [18]. Previous study has associated large vessel diseases with IGFBP-1 abnormalities, suggesting that IGFBP-1 might be a critical factor in the cardiovascular disease pathophysiology [19]. IGFBP1 circulating level was markedly suppressed in dietary-induced obese mice. In ApoE^-/-^ mice, overexpression of IGFBP1 reduced atherosclerosis [19]. Expression of IGFBP-1 was also significantly increased in carotid plaques. Immunohistochemistry demonstrated co-localization of IGFBP-1 with smooth muscle cells (SMCs) and macrophages, and overexpressing IGFBP-1 induced SMC proliferation [20]. However, there is no published paper about the mechanism of IGFBPL1 in atherosclerosis progress and macrophage lipid accumulation. Previous IGFBPL1-related studies were mainly focused on the nervous system and tumorigenesis. In this research, our result showed that IGFBPL1 was reduced in both ox-LDL and PA induced lipid-laden THP-1 macrophages. IGFBPL1 regulates cell IGF signal by binding with IGFs [13]. Recent studies showed that IGFBPL1 bound to IGF-1 and subsequently induced calcium signaling and mammalian target of rapamycin phosphorylation to stimulate axon elongation, which provides a new therapeutic target for promoting axon regeneration and reversing vision loss [21]. Microglia shift toward an inflammatory phenotype during aging-related neurodegeneration. Overexpression of IGFBPL1 in mouse and human microglia resolves lipopolysaccharide-induced neuro-inflammation via activating IGF-1R signaling [22]. In this study, our results also showed that IGFBPL1 regulates lipid accumulation in macrophages in an IGF-1R-dependent manner.

Macrophages plays an important role in process of lipid metabolism in atherosclerosis [3]. Under normal conditions, the uptake and efflux of lipids by macrophages are in a state of dynamic equilibrium [4]. The un-controlled cholesterol uptake by scavenger receptors, such as CD36 and SR-A1, facilitates lipid accumulation in macrophage. In contrast, promotion of ABCA1/ABCG1-mediated lipid efflux reduces the lipid load of macrophages [23]. In this research, we have found that IGFBPL1 only up-regulated ABCG1, but did not change the levels of SR-A, SR-B1, CD36, and ABCA1.

This result suggests that IGFBPL1 may inhibit lipid accumulation in macrophages by promoting ABCG1-mediated cholesterol efflux. ABCG1 belongs to the ABCG subfamily, which accelerates cholesterol efflux from macrophages to mature HDL particles [24]. ABCG1 forms a functional complex by homodimerization or heterodimerization [25]. Expression of ABCG1 is highly up-regulated during macrophage differentiation and cholesterol loading, while ABCG1 is downregulated by cholesterol depletion and statins [26, 27]. Deficiency of ABCG1 disrupts lipid homeostasis and enhances lesion development in mice with LDL receptor knockout [28]. ABCG1 overexpression protected the mice against high fat diet-induced lipid accumulation [29]. In our study, we also have found that IGFBPL1 inhibits THP-1 macrophage lipid accumulation by increasing ABCG1 to promote cholesterol efflux.

ABCA1 is the most important cholesterol transporter under normal physiological conditions [30], but ABCG1 may be a key gene regulating lipid accumulation in cells under certain environment. For instance, PPARγ antagonist GW9662 significantly potentiate mycobacterium tuberculosis induced lipid body formation and inhibit ABCG1 expression, overexpression of ABCG1 significantly reversed the promotion effect of GW9662 on foamy macrophages formation [31]. Metformin decreased ox-LDL-induced cholesterol accumulation and foam cell formation by increasing ABCG1 meditated cholesterol efflux to HDL [32]. Furthermore, LXR activation promotes isotopic cholesterol efflux to HDL particles in macrophages [33], and this induction is almost absent in ABCG1^−/−^ macrophages [29]. In addition, previous studies showed that LXRα independently regulates the expression of ABCA1 or ABCG1 in the presence of specific ligand [34]. Similarly, we also observed that up-regulated LXRα by IGFBPL1 only increased the expression of ABCG1 and had no effect on ABCA1. However, the precise mechanisms of this effect need to be further clarified in the next studies.

In conclusion, IGFBPL1 inhibits macrophage lipid accumulation by enhancing the activation of IGR-1R/LXRα/ABCG1 pathway (Figure 5). Our results provide a theoretical basis of IGFBPL1 in anti-atherosclerosis therapy by reducing lipid accumulation in macrophages.

## MATERIALS AND METHODS

### Cell culture and treatments

The human monocytic cell line THP-1 was purchased from ATCC (TIB-202) and maintained in at 37° C and 5% CO_2_ in RPMI 1640 medium with 10% FBS. The THP-1 macrophages were obtained through treating the THP-1 monocytes with 100 ng/mL phorbol-12-myristate-13-acetate (PMA, MedChemExpress, Monmouth Junction, NJ, USA) for 48 h. Then the cells were transfected with pcDNA3.1 or pcDNA3.1-IGFBPL1 using GP-Transfect-Mate (Jima, Shanghai, China) according the to the instruction. After 24 h of transfection, THP-1 macrophages were treated for 72 h with different reagents.

### Oil red O staining

The THP-1 macrophages were fixed in 4% paraformaldehyde solution (v/v) for 12 min and washed in PBS for 15 s. Then, the cells were stained with filtered oil red O solution at room temperature for 5 min and dispersed with 60% isopropanol solution (v/v) for 10 s. After washing by PBS for 15 s, the cells were stained with hematoxylin solution for 3 min. The images were acquired by Olympus microscope.

### Cellular cholesterol efflux assays

THP-1 macrophage cells were treated with 22-NBD-cholesterol (5 μM) for 4 h. Then, the cells were washed with PBS and transfected with pcDNA3.1-IGFBPL1. After 48 h of transfection, cells were treated with high-density lipoprotein (HDL, 50 μg/mL) for 4 h. The Olympus fluorescence microscope was used to detected the cell fluorescence intensity using 469 nm for excitation and 537 nm for emission.

### Real-time quantitative polymerase chain reaction (qPCR)

Total RNA was extracted through the TRIzol reagent (Invitrogen, Waltham, MA USA) according to the manufacturer’s protocol. The purity and concentration of the extracted RNA was tested by Nanodrop 3000 (Thermo Fisher, Waltham, MA USA). Then, RNA (1 μg) was revers transcription into cDNA using the PrimeScript RT reagent Kit with gDNA Eraser (Takara Bio Inc., Osaka, Japan). qPCR was performed by SYBR Green qPCR Kit (Accurate Biology, Hunan, China). Relative mRNA level was analyzed using the 2^-ΔΔCt^ method and normalized by GAPDH as the internal control. The sequences of the qPCR primers used are shown in Table 1.

**Table 1:** The primer sequence use in this research.

**Gene**	**Forward (5'→3‘)**	**Revise (5'→3‘)**
IGFBPL1	CAGCCAACATGGTGGGAGA	GTCATCGGGAGCTGGGAAG
LXR	TGGAGACATCTCGGAGGTACA	GATAGCAATGAGCAAGGCAA
ABCG1	TCGGTGGATGAGGTGGTGT	CTGGGCTTCCGTGAGGTTATTA
ABCA1	TCAGTGGGATGGATGGCAA	TCCGTCTGGCAATTAGCAGTC
CD36	AAGTGATGATGAACAGCAGC	TCCTCAGCGTCCTGGGTT
SR-A	CACTGATAGCTGCTCCGAATC	ACACGAGGAGGTAAAGGGCA
SR-B1	CAAGGTTGACTTCTGGCATT	AGAACTCCAGCGAGGACTC
GAPDH	ACATCAAGAAGGTGGTGAAG	TCAAAGGTGGAGGAGTGGGT

### Western blot (WB) analysis

THP-1 macrophages were lysed for protein extraction by radio immunoprecipitation assay buffer and phenyl methyl sulfonyl fluoride (Solarbio Life Sciences, Beijing, China). The protein concentration was determined by BCA assay kit (CWBIO, Beijing, China). Proteins (20 μg per lane) were separated with 12% gels using sodium dodecyl sulfate-polyacrylamide gel electrophoresis (80 V, 90 min). Subsequently, proteins were transferred to 0.22 μm polyvinylidene fluoride membranes (Merck Millipore, Darmstadt, Germany). Thereafter, the membranes were blocked by 5% fat-free dry milk dissolved in Tris-buffered saline with Tween-20 at 4° C for 2 h and then immunoblotted with primary antibodies overnight at 4° C. The next day, the membranes were rinsed five times with Tris-buffered saline with Tween-20 (10 min each) and further incubated with horseradish peroxidase-conjugated secondary antibody for 2 h at room temperature. Finally, the protein bands were visualized through enhanced chemiluminescence (Merck Millipore, Darmstadt, Germany), and ImageJ software was used to quantify the relative protein levels. The detailed information of antibody used are shown in Table 2.

**Table 2:** The antibody information in this study.

**Primary antibody**	**Clone**	**Company**	**Catalog no.**	**Dilution**
IGFBPL1	Polyclonal	Affinity	DF2512	1:1000
ABCG1	Monoclonal	Abcam	ab52617	1:2000
LXR	Polyclonal	Bioss	381851	1:700
GAPDH	Monoclonal	CST	2118S	1:1000
**Secondary antibody**	**Conjugate used**	**Company**	**Catalog no.**	**Dilution**
Goat Anti-rabbit IgG	HRP	Bioworld	0295G	1:50000

### Cell counting kit-8 (CCK-8) assay

THP-1 macrophages were seeded in 96-well plates at a concentration of 10,000 cells/well. Cells were cultured with SR9238 or Picropodophyllin (PPP) of different concentrations for 72 h. Then, 10 μL CCK-8 solution was added and incubated cells for 4 h. The microplate reader (Bio Tek, Santa Clara, CA, USA) was used to obtain the absorbance optical density at 450 nm.

### Statistical analysis

All data are presented as the mean ± standard error of the mean (SEM) of three independent experiments. The unpaired Student’s t-test was used for *p*-value calculations by SPSS 22 (IBM Knowledge Center, USA).
